# Screening of traditional Chinese medicines with therapeutic potential on chronic obstructive pulmonary disease through inhibiting oxidative stress and inflammatory response

**DOI:** 10.1186/s12906-016-1347-y

**Published:** 2016-09-13

**Authors:** Ming-Xing Zhou, Xuan Wei, Ai-Ling Li, Ai-Min Wang, Ling-Zi Lu, Yue Yang, Dong-Mei Ren, Xiao-Ning Wang, Xue-Sen Wen, Hong-Xiang Lou, Tao Shen

**Affiliations:** 1 Key Lab of Chemical Biology (MOE), School of Pharmaceutical Sciences, Shandong University, 44 West Wenhua Road, Jinan, 250012 People’s Republic of China; 2 School of Pharmaceutical Sciences, Shandong University of Traditional Chinese Medicine, Jinan, People’s Republic of China

**Keywords:** Traditional Chinese medicines, Chronic obstructive pulmonary disease, Oxidative stress, Inflammatory response

## Abstract

**Background:**

Chronic obstructive pulmonary disease (COPD) is a major public health problem and gives arise to severe chronic morbidity and mortality in the world. Inflammatory response and oxidative stress play dominant roles in the pathological mechanism of COPD, and have been regarded to be two important targets for the COPD therapy. Traditional Chinese medicines (TCMs) possess satisfying curative effects on COPD under guidance of the TCM theory in China, and merit in-depth investigations as a resource of lead compounds.

**Methods:**

One hundred ninety-six of TCMs were collected, and extracted to establish a TCM extract library, and then further evaluated for their potency on inhibitions of oxidative stress and inflammatory response using NADP(H):quinone oxidoreductase (QR) assay and nitric oxide (NO) production assay, respectively.

**Results:**

Our investigation observed that 38 of the tested TCM extracts induced QR activity in hepa 1c1c7 murine hepatoma cells, and 55 of them inhibited NO production in RAW 264.7 murine macrophages at the tested concentrations. Noteworthily, 20 of TCM extracts simultaneously inhibited oxidative stress and inflammatory responses.

**Conclusion:**

The observed bioactive TCMs, particularly these 20 TCMs with dual inhibitory effects, might be useful for the treatment of COPD. More importantly, the results of the present research afford us an opportunity to discover new lead molecules as COPD therapeutic agents from these active TCMs.

**Electronic supplementary material:**

The online version of this article (doi:10.1186/s12906-016-1347-y) contains supplementary material, which is available to authorized users.

## Background

Chronic obstructive pulmonary disease (COPD) is a disease characterized by progressive and not fully reversible airflow limitation, which is associated with abnormal inflammatory response of the lung to noxious particles and gases [1]. Tobacco smoke, indoor and outdoor air pollutions, as well as exposure to occupational dust and chemicals are the three dominant risk factors for COPD. It is the fourth leading cause of chronic morbidity and mortality in the United States. On the basis of investigation by the World Bank/World Health Organization, COPD is predicted to rank fifth in 2020 as a worldwide burden of disease. A horrifying fact is that half of global deaths from COPD occur in the Western Pacific Region, with the majority of these existing in China, which might be contributing to high incidence of smoking and severe air pollution in the industrialization advancement [2].

Cumulative evidences indicate that inflammatory response, oxidative stress, and protease imbalance play dominant roles in the pathological mechanism of COPD [3, 4]. Briefly, exogenous irritants and reactive oxygen species (ROS) activate inflammatory cells (e.g. macrophages, neutrophils) and epithelial cells in the respiratory tract that release ROS, inflammatory mediators [e.g. leukotriene B4 (LTB4), interleukin-8 (IL-8), tumor necrosis factor α (TNFα), transforming growth factor-β (TGF-β)], proteases (e.g. cathepsins, matrix metalloproteinases)[3, 5, 6]. ROS stimulates nuclear factor kB (NF-kB) and increase the release of inflammatory cytokines, inflammatory mediators promote the production of endogenous ROS, while proteases cause alveolar destruction and mucus secretion. Hence, the synergistic reactions of inflammation, oxidative stress, and protease imbalance amplify pathophysiology of COPD, and inhibitions of these three processes are regarded to be effective strategies for the treatment, as well as drug research and development of COPD [7].

Plenty of traditional Chinese medicines (TCMs) have been used clinically to treat COPD in the form of single or compound prescription under guidance of the TCM theory in China, and demonstrated satisfying curative effects [8, 9]. Their clinical effectiveness implies that TCM is an important resource of new drugs and/or lead compounds with COPD therapeutic potential. Based on this rationale, we have launched a systemic research on discovering new drugs and lead molecules for COPD treatment from TCM targeting inhibitions of oxidative stress and inflammatory response. We firstly collected and extracted TCM materials to establish a TCM extract library, and then carried out a biological screening of these TCMs using NADP(H):quinone oxidoreductase (QR) assay and nitric oxide (NO) production assay to find the TCMs with potential therapeutic effect on COPD.

## Methods

### Chemicals

Sulforaphane (SF, purity >98 %) was purchased from Sigma-Aldrich (St. Louis, MO, USA). Didox (purity >98 %) was purchased from MedChem Express (Monmouth Junction, ON, USA). Solvents used for extraction were of analytical grade and obtained from Tianjin Fuyu Chemical Company (Tianjin, China).

### Collection and Identification of tested TCMs

Traditional Chinese medicine (TCM) materials were purchased from the Jinan Jianlian TCM Co. Ltd in Shandong province, Anguo TCM market in Hebei province, and Bozhou TCM market in Anhui Province. These TCMs were identified by Prof. Lan Xiang, School of Pharmaceutical Sciences, Shandong University, through comparing their characteristics in plant morphology and taxonomy with that described in Chinese Pharmacopoeia. Voucher specimens (Voucher ID see Table 1) of TCMs have been deposited at the Laboratory of Pharmacognosy, School of Pharmaceutical Sciences, Shandong University.

**Table 1:** Inhibitions on oxidative stress and inflammation of TCMs evaluated using QR induction and NO production assay

No	Plant name	Part used in TCM	Voucher ID	Yields (%)	Induction of QR activity (MQI)	Inhibition of NO production (MIR)
1	*Acacia catechu* (L.f.) Wild.	Branch	20151128-100-EC	69.8	N/D	N/D
2	*Acanthopanax gracilistylus* W. W. Smith	Root-bark	20150802-20-WJP	9.8	N/D	68.0 % (100)
3	*Acanthopanax senticosus* (Rupr. et Maxim.) Harms	Rhizome	20150801-8-CWJ	7.7	N/D	52.0 % (200)
4	*Achyranthes bidentata* Bl.	Rhizome	20150801-4-NX	8.7	N/D	N/D
5	*Aconitum carmichaeli* Debx.	Root	20151128-58-FZ	14.7	N/D	N/D
6	*Acorns tatarinowii* Schott	Rhizome	20151128-30-SCP	10.5	N/D	N/D
7	*Adenophora tetraphylla* (Thunb.) Fisch.	Root	20151128-76-SS	19.1	N/D	N/D
8	*Agrimonia pilosa* Ledeb.	Aerial part	20151128-31-XHC	9.8	N/D	41.2 % (200)
9	*Akebia trifoliata* (Thunb.) Koidz. subsp. *australis* (Diels) T. Shimizu	Rattan	20150802-14-BMT	11.6	N/D	N/D
10	*Albizia julibrissin* Durazz.	Bark	20151128-134-HHP	8.9	1.64 fold (200)	N/D
11	*Alisma orientalis* (Sam.) Juzep.	Root	20151128-71-ZX	13.6	N/D	34.2 % (200)
12	*Allium tuberosum* Rottl.	Seed	20150717-4-JCZ	3.8	N/D	N/D
13	*Amomum kravanh* Pierre ex Gagnep.	Fruit	20151128-137-DK	2.1	N/D	N/D
14	*Amomum villosum* Lour.	Fruit	20150801-9-SR	8.3	N/D	N/D
15	*Ampelopsis japonica* (Thunb.) Makino	Root	20151128-127-BL	10.7	N/D	N/D
16	*Andrographis paniculata* (Burm.f.) Nees	Aerial part	20151128-83-CXL	9.9	2.04 fold (200)	N/D
17	*Anemarrhena asphodeloides* Bge.	Rhizome	20150802-17-ZM	10.2	N/D	41.2 % (200)
18	*Angelica dahurica* (Fisch. ex Hoffm.) Benth. et Hook.f.	Root	20151128-125-BZ	8.9	N/D	N/D
19	*Angelica pubescens* Maxim. f. *biserrata* Shan et Yuan	Root	20150802-16-DH	25.1	N/D	N/D
20	*Angelica sinensis* (Oliv.) Diels	Root	20151128-34-DG	19.4	1.41 fold (200)	N/D
21	*Arctium lappa* L.	Fruit	20151128-33-NBZ	18.4	N/D	N/D
22	*Areca catechu* L.	Peel	20151128-104-DFP	2.3	N/D	N/D
23	*Areca catechu* L.	Fruit	20151128-145-BL	8.2	1.33 fold (25)	N/D
24	*Arisaema erubescens* (Wall.) Schott	Tuber	20151128-23-TNX	1.2	1.36 fold (200)	N/D
25	*Aristolochia debilis* Sieb. et Zucc.	Aerial part	20151128-21-TXT	3.2	N/D	N/D
26	*Artemisia argyi* Levi. et Vant.	Leaf	20150716-6-AY	12.9	N/D	86.2 % (200)
27	*Artemisia scoparia* Waldst. et Kit.	Aerial part	20151128-74-YC	12.8	1.48 fold (200)	38.8 % (100)
28	*Asarum heterotropoides* Fr. Schmldt var. *mandshuricum* (Maxim.) Kitag.	Root and rhizome	20151128-72-XX	11.4	N/D	N/D
29	*Atractylode lancea* (Thunb.) DC.	Rhizome	20151128-136-CZ	24.2	N/D	52.3 %(200)
30	*Atractylodes macrocephala* Koldz.	Rhizome	20151128-123-BZ	12.9	N/D	N/D
31	*Aucklandia lappa* Decne.	Root	20151128-15-MX	16.3	2.31 fold (25)	94.7 % (25)
32	*Belamcanda chinensis* (L.) DC.	Rhizome	20151128-106-SG	29.6	N/D	N/D
33	*Bletilla striata* (Thunb.) Reichb.f.	Rhizome	20150801-12-BJ	2.1	N/D	N/D
34	*Bolbostemma paniculatum* (Maxim.) Franquet	Tuber	20151128-5-TBM	15.1	N/D	N/D
35	*Callicarpa macrophylla* Vahl	Leaf	20151128-7-DYZZ	4.3	N/D	85.8 % (200)
36	*Cassia angustifolia* Vahl.	Leaf	20150802-25-FXY	9.4	1.54 fold (50)	79.7 % (200)
37	*Cassia obtusifolia* L.	Seed	20151128-38-JMZ	7.9	N/D	N/D
38	*Chaenomeles speciosa* (Sweet) Nakai	Fruit	20151128-24-MG	30.5	N/D	N/D
39	*Chrysanthemum morifolium* Ramat.	Flower	20150802-28-JH	23.3	N/D	90.2 % (200)
40	*Cimicifuga heracleifolia* Kom.	Rhizome	20151128-28-SM	14.1	1.95 fold (100)	86.4 % (200)
41	*Cinnamomum cassia* Presl	Branch	20150717-2-GZ	5.0	N/D	N/D
42	*Cirsium japonicum* Fisch. ex DC.	Aerial part	20151128-9-DJ	6.7	N/D	N/D
43	*Cirsium setosum* (Willd.) M.Bieb.	Aerial part	20151128-10-XJ	12.7	1.51 fold (200)	38.8 % (200)
44	*Cistanchede deserticola* Y. C. Ma	Stem	20150801-7-RCR	23.6	N/D	N/D
45	*Citrus aurantium* L.	Fruit	20150801-11-ZQ	17.0	N/D	N/D
46	*Citrus limon* (L.) Burm. f.	Fruit	20150716-16-NM	19.5	1.79 fold (200)	N/D
47	*Citrus medica* L. var. *sarcodac-tylis* Swingle	Fruit	20151128-55-FS	39.6	N/D	N/D
48	*Citrus reticulata* Blanco.	Pericarp	20150716-10-CP	22.0	N/D	N/D
49	*Clematis armandii* Franch.	Rattan	20150802-12-CMT	4.5	N/D	N/D
50	*Codonopsis pilosula*(Franch.) Nannf.	Root	20151128-59-DS	48.4	N/D	N/D
51	*Coix lacryma-jobi* L. var. *ma-yuen* (Roman.) Stapf	Seed	20150716-15-YYR	6.5	N/D	N/D
52	*Commelina communis* L.	Aerial part	20151128-88-YZC	4.4	N/D	N/D
53	*Coptis chinensis* Franch.	Rhizome	20150802-31-HL	6.6	N/D	57.1 %(200)
54	*Cornus officinalis* Sieb. et Zucc.	Fruit	20150802-27-SZY	43.6	N/D	N/D
55	*Crataegus pinnatifida* Bge. var. *major* N. E. Br.	Fruit	20150716-2-SZ	34.8	N/D	N/D
56	*Cremastra appendiculata* (D. Don) Makino	Pseudobulb	20151128-12-SCG	2.6	N/D	61.0 % (200)
57	*Croton tiglium* L.	Fruit	20150802-8-BD	1.1	N/D	N/D
58	*Curculigo orchioides* Gaertn.	Rhizome	20151128-121-XM	4.5	1.57 fold (200)	47.8 % at (200)
59	*Curcuma phaeocaulis* Val.	Rhizome	20150801-20-EZ	2.6	N/D	66.9 % (25)
60	*Curcuma wenyujin* Y. H. Chen et C. Ling	Root	20150802-26-YJ	9.0	N/D	N/D
61	*Cynanchum atratum* Bge.	Root and rhizome	20151128-40-BW	23.6	N/D	N/D
62	*Cynanchum stauntonii* (Decne.) Schltr ex Lévl.	Rhizome	20150801-13-BQ	10.6.	N/D	N/D
63	*Cynomorium songaricum* Rupr.	Stem	20150716-14-SY	17.9	N/D	N/D
64	*Cyperus rotundus* L.	Rhizome	20151128-80-XF	11.6	1.74 fold (200)	N/D
65	*Dendrobium nobile* Lindl.	Stem	20150802-13-MH	9.0	N/D	38.9 % (200)
66	*Dictamnus dasycarpus* Turcz.	Velamen	20150802-4-BXP	9.0	N/D	N/D
67	*Dioscorea opposita* Thunb.	Rhizome	20150716-1-SY	1.7	N/D	N/D
68	*Dipsacus asperoides* C. Y. Cheng et T. M. Ai	Rhizome	20150716-13-XD	17.5	N/D	38.6 % (200)
69	*Drynaria fortunei* (Kunze) J. Sm.	Rhizome	20151128-78-GSB	4.8	N/D	N/D
70	*Eclipta prostrata* L.	Aerial part	20151128-99-MHL	9.0	N/D	N/D
71	*Epimedium brevicornum* Maxim.	Aerial part	20150716-9-YYH	20.5	N/D	N/D
72	*Equisetum hiemale* L.	Aerial part	20151128-108-MZ	4.9	N/D	41.7 %(200)
73	*Eriocaulon buergerianum* Koern.	Flower	20151128-139-GJC	7.9	N/D	N/D
74	*Eucommia ulmoides* Oliv.	Root-bark	20151128-51-DZ	8.3	1.56 fold (200)	62.5 % (200)
75	*Eugenia caryophyllata* Thunb.	Bud	20151128-1-DX	27.5	N/D	N/D
76	*Eupatorium fortunei* Turcz.	Aerial part	20151128-66-PL	10.1	N/D	N/D
77	*Euphorbia humifusa* Willd.	Whole plant	20151128-37-DJC	9.5	N/D	N/D
78	*Ferula Sinkiangensis* K. M. Shen	Resin	20151128-140-EW	6.1	N/D	N/D
79	*Forsythia suspense* (Thnub.) Vahl	Fruit	20151128-53-LQ	28.3	N/D	48.3 % (200)
80	*Fraxinus rhynchophylla* Hance	Bark	20151128-86-QP	8.0	N/D	90.7 % (200)
81	*Fritillaria ussuriensis* Maxim.	Bulb	20151128-118-PBM	4.1	N/D	N/D
82	*Ganoderma sinense* Zhao, Xu et Zhang	Sporophore	20150801-2-ZZ	2.9	N/D	N/D
83	*Gardenia jasminoides* Ellis.	Fruit	20150717-7-ZZ	16.1	N/D	N/D
84	*Gastrodia elata* Bl.	Tuber	20150801-16-TM	7.5	N/D	N/D
85	*Glycyrrhiza uralensis* Fisch.	Rhizome	20150716-5-GC	15.7	2.19 fold (100)	82.9 % (200)
86	*Hippophae rhamnoides* L.	Fruit	20151128-57-SJ	36.5	N/D	N/D
87	*Homalomena occulta* (Lour.) Schott	Rhizome	20151128-13-QNJ	9.3	N/D	N/D
88	*Hordeum vulgar*e L.	Fruit	20151128-135-MY	11.5	N/D	N/D
89	*Houttuynia cordata* Thunb.	Aerial part	20150801-18-YXC	16.7	N/D	N/D
90	*Illicium difengpi* K. I .B. et K. I. M.	Bark	20151128-132-DFP	1.9	1.52 fold (100)	N/D
91	*Illicium verum* Hook. f.	Fruit	20151128-2-BJHX	13.3	N/D	55.7 % (200)
92	*Inula helenium* L.	Root	20150802-5-TMX	13.2	1.77 fold (12.5)	100 % (100)
93	*Isatis indigotica* Fort.	Root	20150802-9-BLG	17.9	N/D	N/D
94	*Isatis indigotica* Fort.	Leaf	20151128-102-DQY	13.6	1.66 fold (50)	N/D
95	*Kaempferia galanga* L.	Rhizome	20151128-105-SN	4.7	N/D	N/D
96	*Kochia scoparia* (L.) Schrad.	Fruit	20150802-23-DFZ	8.5	N/D	N/D
97	*Laminaria Japonica* Aresch.	Thallus	20150802-10-KB	18.5	N/D	N/D
98	*Lepidium apetalum* Willd.	Seed	20150802-22-TLZ	2.9	N/D	N/D
99	*Ligusticum chuanxiong* Hort.	Rhizome	20151128-19-CX	16.1	1.73 fold (200)	69.0 % (100)
100	*Ligustrum lucidum* Ait.	Fruit	20151128-18-NZZ	24.2	N/D	N/D
101	*Lilium lancifolium* Thunb.	Leaf	20151128-32-BH	4.3	N/D	N/D
102	*Lindera aggregata* (Sims) Kosterm.	Root	20150801-15-WY	10.7	1.59 fold (200)	N/D
103	*Lithospermum erythrorhizon* Sieb. et Zucc.	Root	20151128-93-ZC	6.4	1.52 fold (50)	57.1 % (200)
104	*Lobelia chinensi* Lour.	Whole plant	20151128-129-BBL	23.7	N/D	N/D
105	*Lonicera hypoglauca* Miq.	Flower	20150801-1-SYH	31.4	N/D	N/D
106	*Lonicera japonica* Thunb.	Flower	20150801-3-JYH	26.7	N/D	N/D
107	*Lophatherum gracile* Brongn.	Stem and leaf	20151128-91-DZY	8.6	N/D	N/D
108	*Lycium barbarum* L.	Fruit	20150801-6-GQ	11.5	N/D	N/D
109	*Lycium chinense* Mill.	Root-bark	20150802-21-DGP	7.5	N/D	N/D
110	*Lycopodium japonicum* Thunb.	Whole plant	20151128-138-SJC	22.3	N/D	53.0 % (200)
111	*Lycopus lucidus*Turcz. var. *hirtus* Regel	Aerial part	20151128-70-ZL	13.5	N/D	61.6 % (200)
112	*Lysimachia christinae* Hance	Whole plant	20151128-67-JQC	11.2	N/D	N/D
113	*Mahonia bealei* (Fort.) Carr.	Stem	20151128-114-GLM	6.1	N/D	N/D
114	*Melia toosendan* Sleb. et Zucc.	Fruit	20151128-107-CLZ	16.2	N/D	N/D
115	*Menispermum dauricum* DC.	Rhizome	20151128-120-BDG	11.0	N/D	N/D
116	*Mentha haplocalyx* Briq.	Aerial part	20150716-8-BH	19.1	N/D	N/D
117	*Mignolia officinalis* Rehd. et Wils.	Bark	20151128-84-HP	22.9	N/D	N/D
118	*Misla chinensis* Maxim.	Aerial part	20151128-81-XR	6.9	1.60 fold (100)	N/D
119	*Morinda officinalis* How.	Root	20151128-112-BJT	28.8	N/D	N/D
120	*Morus alba* L.	Branch	20151128-142-SZ	7.4	1.37 fold (100)	61.2 % (200)
121	*Morus alba* L.	Fruit	20151128-143-SS	29.9	N/D	N/D
122	*Nardostachys chinensis* Batal.	Root and rhizome	20151128-115-GS	12.3	N/D	N/D
123	*Oroxylum inddicum* (L.) Vent.	Seed	20151128-26-MHD	14.0	N/D	85.4 % (200)
124	*Orostachys fimbriatus* (Turcz.) Berg.	Aerial part	20151128-109-WS	6.3	N/D	N/D
125	*Paeonia lactiflora* Pall.	Rhizome	20150716-7-BS	6.4	N/D	N/D
126	*Panax ginseng* C. A. Mey	Rhizome	20150801-10-SSS	39.4	N/D	N/D
127	*Panax quinque folium* L.	Root	20150802-29-XYS	14.8	N/D	N/D
128	*Perilla frutescens* (L.) Britt.	Leaf	20150717-6-ZS	2.8	1.73 fold (200)	57.8 % (200)
129	*Peucedanum praeruptorum* Dunn	Root	20151128-85-QH	20.6	N/D	77.6 % (200)
130	*Phellodendron chinense* Schneid.	Tree-bark	20151128-41-HB	12.7	N/D	N/D
131	*Phragmites communis* Trin.	Root	20151128-49-LG	3.8	N/D	N/D
132	*Physalis alkekengi* L. var. *franchetii* (Mast.) Makino	Calyx	20150730-2-GJD	14.2	1.79 fold (200)	91.4 % (200)
133	*Pinellia ternate* (Thunb.) Breit.	Tuber	20151128-130-BX	0.8	1.74 fold (200)	N/D
134	*Piper nigrum* L.	Fruit	20151128-92-HHJ	5.4	N/D	N/D
135	*Plantago asiatica* L.	Whole plant	20150716-4-CQC	13.7	N/D	N/D
136	*Platycodon grandiflorum* (Jacq.) A. DC.	Root	20151128-87-JG	33.4	N/D	N/D
137	*Pogostemon cablin* (Blanco) Benth.	Aerial part	20151128-16-GHX	5.5	1.73 fold (100)	56.6 % (200)
138	*Polygonatum kingianum* Coll. et Hemsl.	Rhizome	20151128-90-HJ	6.8	N/D	N/D
139	*Polygonatum odoratum* (Mill.) Druce	Rhizome	20151128-113-YZ	21.6	N/D	N/D
140	*Polygonum aviculare* L.	Aerial part	20151128-89-BX	10.8	N/D	N/D
141	*Polygonum cuspidatum* Sieb. et Zucc.	Root and rhizome	20151128-63-HZ	21.7	N/D	60.5 % (200)
142	*Polygonum multiflorum* Thunb.	Root	20151128-54-HSW	6.8	N/D	35.5 % (200)
143	*Polyyala tenuifolia* Willd.	Root	20151128-46-YZ	34.4	N/D	N/D
144	*Potentilla chinensis* Ser.	Whole plant	20151128-65-WLC	8.9	N/D	N/D
145	*Prunella vulgaris* L.	Peel	20150716-11-XKC	4.1	N/D	N/D
146	*Prunus armeniaca* L. var. *ansu* Maxim.	Seed	20151128-60-KXR	5.7	N/D	N/D
147	*Prunus persica* (L.) Batsch	Seed	20151128-61-TR	3.7	N/D	N/D
148	*Pseudolarx kaempleri* Gord.	Velamen	20151128-6-TJP	14.8	N/D	N/D
149	*Pseudostellaria beterphylla* (Miq.) Pax ex Pax et Hoffm.	Root	20151128-27-TZS	13.5	N/D	N/D
150	*Psoralea corylifolia* L.	Fruit	20150801-19-BGZ	10.2	N/D	N/D
151	*Pulsatilla chinensis* (Bunge) Regel	Root	20151128-124-BTW	22.7	N/D	N/D
152	*Punica granatum* L.	Peel	20151128-117-SLP	31.3	N/D	N/D
153	*Pyrrosia sheareri* (Bak.) Ching	Leaf	20151128-116-SW	12.2	1.85 fold (50)	N/D
154	*Rabdosia rubescens* (Hemsl.) Hara	Aerial part	20151128-39-DLC	8.4	1.38 fold (100)	43.2 % (200)
155	*Raphanus sativus* L.	Seed	20150802-30-LFZ	13.8	N/D	N/D
156	*Rhaponlicum uniflorum* (L.) DC.	Root	20151128-97-LL	4.4	1.54 fold (200)	N/D
157	*Rheum palmatum* L.	Root and rhizome	20151128-103-DH	25.6	N/D	60.2 % (200)
158	*Rhodiola crenulata* (Hook. f. et Thoms.) H. Ohba	Rhizome	20150801-5-HJT	13.3	N/D	46.3 % (200)
159	*Rosa chinensis* Jacq.	Flower	20151128-110-YJH	18.9	N/D	N/D
160	*Rosa laevigata* Michx.	Fruit	20151128-68-JYZ	25.2	1.67 fold (50)	57 % (200)
161	*Rubia cordifolia* L.	Root and rhizome	20151128-73-QC	10.0	N/D	N/D
162	*Salvia miltiorrhiza* Bge.	Root and rhizome	20151128-29-DS	39.7	1.44 fold (100)	64.5 % (200)
163	*Sanguisorba officinalis* L.	Root	20151128-36-DY	3.5	N/D	N/D
164	*Saposhnikovia divaricata* (Turcz.) Schischk.	Root	20150802-19-FF	15.9	1.95 fold (100)	N/D
165	*Sareassum pallidum* (Turn.) C. Ag.	Frond	20150802-1-HZ	11.5	N/D	N/D
166	*Sargentodoxa cuneate* (Oliv.) Rehd. et Wils.	Rattan	20151128-8-DXT	16.9	N/D	N/D
167	*Scrophularia ningpoensis* Hemsl.	Root	20151128-128-XS	50.5	N/D	N/D
168	*Scutellaria baicalensis* Georgi.	Rhizome	20150716-12-HQ	30.2	N/D	87.4 %(200)
169	*Scutellaria barbata* D. Don	Whole plant	20151128-35-BZL	10.2	N/D	59.0 % (200)
170	*Sedum sarmentosum* Bunge.	Whole plant	20151128-64-CPC	17.5	N/D	N/D
171	*Selaginella tamariscina* (Beauv.) Spring	Whole plant	20151128-69-JB	8.9	N/D	N/D
172	*Senecio scandens* Buch.-Ham.	Aerial part	20151128-14-QLG	11.4	N/D	38.1 % (200)
173	*Sesamum indicum* L.	Seed	20150717-3-HZM	3.3	N/D	N/D
174	*Siegesbeckia orientalis* L.	Aerial part	20151128-98-XXC	4.8	1.91 fold (200)	54.9 % (200)
175	*Siphonostegia chinensis* Benth.	Whole plant	20151128-119-BLJM	9.7	N/D	34.6 % (200)
176	*Smilax glabra* Roxb.	Rhizome	20151128-101-TFL	10.9	N/D	N/D
177	*Sophora flavescens* Ait.	Root	20151128-62-KS	21.4	N/D	N/D
178	*Sophora japonica* L.	Flower and bud	20151128-95-HH	37.1	1.44 fold (200)	N/D
179	*Sparganium stoloniferum* Buch.-Ham.	Tuber	20151128-3-SL	4.5	1.57 fold (200)	N/D
180	*Spatholobus suberectus* Dunn	Rattan	20151128-56-JXT	5.8	N/D	N/D
181	*Stemona sessilifolia* (Miq.) Miq.	Root	20150802-24-BB	30.6	N/D	N/D
182	*Stephania tetrandra* S. Moore	Root	20151128-43-FJ	72.9	N/D	50.0 % (200)
183	*Sterculia lychnophora* Hance	Seed	20150801-21-PDH	3.2	N/D	30.7 % (200)
184	*Taraxacum mongolicum* Hand.-Mazz.	Whole plant	20150716-3-PGY	17.2	N/D	42.3 % (200)
185	*Taxillus chinensis* (DC.) Danser	Aerial part	20150802-15-SJS	6.3	N/D	N/D
186	*Trichosanthes kirilowii* Maxim.	Seed	20131120-1-GL	20.5	N/D	N/D
187	*Tripterygium wilfordii* Hook. f.	Root	20150802-18-LGT	8.3	N/D	N/D
188	*Tussilago farfara* L.	Bud	20150802-6-KDH	9.9	2.38 fold (50)	N/D
189	*Uncaria rhynchophylla* (Miq.) Miq. ex Havil.	Stem	20151128-79-GT	6.5	N/D	N/D
190	*Usnea diffracta* Vain.	Thallus	20150802-2-SL	11.0	N/D	44.5 % (200)
191	*Vaccaria segetatis* (Neck.) Garcke	Seed	20151128-20-WBLX	4.9	N/D	N/D
192	*Verbena officinalis* L.	Aerial part	20151128-17-MBC	7.9	N/D	N/D
193	*Viola yedoensis* Makino	Whole plant	20150802-7-ZHDD	25.6	N/D	N/D
194	*Xanthium sibiricum* Patr.	Fruit	20151128-47-CEZ	3.5	N/D	N/D
195	*Zanthoxylum nitidum* (Roxb.) DC.	Root	20151128-52-LMZ	6.1	N/D	38.2 % (200)
196	*Zanthoxylum schinifolium*Sleb. et Zucc.	Peel	20151128-48-HJ	21.2	1.75 fold (200)	50.3 % (200)

SF (2.0 μM) with an approximately 1.7-fold induction was used as a positive control for QR assay; Didox (100 μM) with an approximately 60 % inhibition of NO production was adopted as a positive control for NO inhibitory assay; MQI: the maximum folds of QR inducing activity under the tested concentration; MIR: the maximum inhibition rate of NO production under the nontoxic tested concentration; N/D, undetected

### Preparations of TCM extractions

Crushed aerial parts or leaves of plant materials (50 g) were extracted under reflux for 2 h with 75 % ethanol (EtOH, 2 × 500 mL), and then EtOH was removed under reduced pressure. The yield of each extract was presented as a percentage of weight of dried plant material, and has been summarized in Table 1.

### Cell cultures

Hepa 1c1c7 murine hepatoma cells (American Type Culture Collection, ATCC) were maintained in Eagle’s minimal essential medium (MEM, Gibco) supplemented with 10 % fetal bovine serum (FBS, Gemini Bio-product). RAW 264.7 murine macrophages (ATCC) were cultured in Dulbecco’s Modified Eagle’s Medium (DMEM, Gibco) supplemented with 10 % FBS. All cells were incubated at 37 °C in a humidified incubator containing 5 % CO_2_.

### NADP(H): quinone oxidoreductase (QR) assay

NADP(H):quinone oxidoreductase (QR) assay was modified from previously described method [10]. Hepa 1c1c7 cells (1.0 × 10^4^ cells/well) were seeded in 96-well plates and treated with the indicated doses of tested extracts for 24 h. The medium was decanted, and the cells were incubated with 40 μL of lysing solution [0.8 % digitonin and 2 mM EDTA solution (pH 7.8)] for 15 min at 37 °C. Then, 170 μL of a complete reaction mixture containing bovine serum albumin, 3-(4,5-dimethylthiazol-2-yl)-2,5-diphenyltetrazoliumbromide (MTT), 1.5 % Tween 20, 0.5 M Tris–HCl, 7.5 mM flavin adenine dinucleotide (FAD), 150 mM glucose-6-phosphate, 10 units/μL glucose-6-phosphate dehydrogenase, 50 mM NADP, and 50 mM menadione was added into each well. After incubation for 4 min, a blue color was developed and the reaction was arrested by adding 50 μL per well of a 0.3 mM dicoumarol solution (pH 7.4). Absorbance was measured at 630 nm on the Model 680 plate reader (Bio-rad). SF (2.0 μM) was adopted as a positive control.

### Nitric oxide (NO) production assay

Inhibition of NO production by LPS-stimulated RAW 264.7 murine macrophages was applied to evaluate anti-inflammatory functions of TCM extracts. RAW 264.7 cells (8.0 × 10^4^ cells/well) were seeded in 96-well plates and treated with 1 μg/mL LPS, in the absence or presence of tested TCM extractions for 24 h. Then, 100 μL of supernatant was removed to a new 96-well plate and added with 100 μL of Griess reagent (0.1 % naphthylethylenediamine and 1 % sulfanilamide in 5 % H_3_PO_4_ solution) at room temperature for 15 min. Absorbance was measured at 570 nm on the Model 680 plate reader (Bio-rad). Nitrite concentration was calculated from a NaNO_2_ standard curve. Didox (100 μM) was used as a positive control.

### Cell viability assay

The anti-proliferative effect of TCM extracts on RAW 264.7 cells were simultaneously determined using a 3-(4,5-dimthylthiazol-2-yl)-2,5-diphenyltetrazolium bromide (MTT, Sigma) assay. Briefly, 100 μL of DMEM media containing 0.4 % MTT were added to each wells, after removing 100 μL of supernatant as described in NO production assay. Then, the cells were incubated at 37 °C for 3 h, and absorbance was measured at 570 nm on the Model 680 plate reader (Bio-rad).

### Statistical analysis

One way analysis of variance (ANOVA) and post hoc multiple comparison Bonferroni test were applied to compare the significant difference between two groups. Results are presented as the mean ± SD. *P* < 0.05 was considered to be significant.

## Results and Discussion

To establish a TCM extract library for biological screening, we firstly selected 196 TCMs based on Chinese Pharmacopoeia (Edition 2015) and TCM literatures, and collected TCMs from Jinan local TCM drugstore, as well as the two biggest Chinese TCM markets, Anguo and Bozhou TCM markets. After plant material authentication, TCMs were extracted with 75 % EtOH to prepare their EtOH extracts, and then the concentrations of 200, 100, 50, 25, 12.5, 6.25 μg/mL were selected as tested doses for bioassays. Names, origin, extract yields and biological activities of investigated TCMs were summarized in Table 1.

We adopted a bioassay measuring QR activity in hepa 1c1c7 murine hepatoma cells to evaluate the ability of TCM extracts on inhibiting of oxidative stress [10]. Although QR is a phase II detoxification enzyme, it possesses same regulating mechanism with antioxidant enzymes [e.g. glutamate-cysteine ligase, modifier subunit (GCLM) and heme oxygenase-1 (HO-1)], since these enzymes are antioxidant response element (ARE)-containing target genes and are mediated by ARE located in their promoter region [11]. Specially, upon the exposure of cells to oxidative stress and/or toxicants, nuclear factor E2-related factor 2 (Nrf2) translocates into the nucleus, binds to the ARE sequence, and activates the transcription of these ARE-target genes [12]. Therefore, QR and antioxidant enzymes (e.g. GCLM and HO-1) possess same responses against endogenous and exogenous insults, which have also been verified by our recent researches [13, 14]. Considering above mentioned inducing mechanism of QR and antioxidant enzymes, determination of QR activity is a rational and effective method for analyzing the potency of oxidative stress inhibition. In the current study, we normalized the data by setting the untreated control group as 1, and then the QR inducing activity of tested extracts was represented by the maximum folds of QR inducing activity (MQI) compared with the untreated control group. SF as a positive control displayed an approximately 1.7-fold induction at 2.0 μM. 1.3-fold of QR inducing activity (MQI = 1.3) under the tested concentrations was adopted as a criterion for bioactive TCM extracts. To be more precise, the level of QR inducing activity was ranked as the following criteria: strong (MQI ≥ 1.8); moderate (1.8 > MQI ≥1.5); weak (1.5 > MQI ≥1.3); undetected (MQI < 1.3).

Ultimately, 38 TCM extracts demonstrated the QR inducing activities with MQI ranging from 1.33- to 2.38- folds under the tested concentrations (Table 1). Of which, eight TCM extracts strongly induced QR activity in hepa 1c1c7 cells (MQI ≥ 1.8), including *Andrographis paniculata* (aerial part, 16), *Aucklandia lappa* (root, 31), *Cimicifuga heracleifolia* (rhizome, 40), *Glycyrrhiz uralensis* (rhizome, 85), *Pyrrosia sheareri* (leaf, 153), *Saposhnikovia divaricate* (root, 164), *Siegesbeckia orientalis* (aerial part, 174), and *Tussilago farfara* (bud, 188). Twenty-two extracts are moderate QR inducers (1.8 > MQI ≥1.5), containing *Albizia julibrissin* (bark, 10), *Cassia angustifolia* (leaf, 36), *Cirsium setosum* (aerial part, 43), *Citrus limon* (fruit, 46), *Curculigo orchioides* (rhizome, 58), *Cyperus rotundus* (rhizome, 64), *Eucommia ulmoides* (root-bark, 74), *Illicium difengpi* (bark, 90), *Inula helenium* (root, 92), *Isatis indigotica* (leaf, 93), *Ligusticum chuanxiong* (rhizome, 99), *Lindera aggregate* (root, 102), *Lithospermum erythrorhizon* (root, 103), *Misla chinensis* (aerial part, 118), *Perilla frutescens* (leaf, 128), *Physalis alkekengi* L. var. *franchetii* (calyx, 132), *Pinellia ternata* (tuber, 133), *Pogostemon cablin* (aerial part, 137), *Rhaponlicum uniflorum* (root, 156), *Rosa laevigata* (fruit, 160), *Sparganium stoloniferum* (tuber, 179), and Z*anthoxylum schinifolium* (peel, 196). Moreover, eight extracts possessed weak QR inducing effect (1.5 > MQI ≥1.3), including *Angelica sinensis* (root, 20), *Areca catechu* (fruit, 23), *Arisaema erubescens* (tuber, 24), *Artemisia scoparia* (aerial part, 27), *Morus alba* (branch, 120), *Rabdosia rubescens* (aerial part, 154), *Salvia miltiorrhiza* (root and rhizome, 162), and *Sophora japonica* (flower and bud, 178). QR inducing effects of 38 bioactive TCM extracts in hepa 1c1c7 cells have been detailedly summarized in Additional file 1: Table S1 and Figure S1.

During the chronic inflammation process, excessive NO have been produced and involved in the tissue injury through damages to proteins, lipids, DNA, and the modulation of leukocyte activity [15]. Accordingly, inhibiting NO production is regarded to be an effective strategy for the therapy of inflammation-related diseases. Herein, we detected NO level in LPS-stimulated RAW264.7 macrophages to evaluate anti-inflammatory function of TCM extracts. Cytotoxicities of tested TCM extracts were simultaneously evaluated by a MTT assay to confirm that the decrease of NO production was not attributed to inhibition of cell proliferation. The maximum inhibition rate (MIR) of NO production under the nontoxic tested concentration, which was calculated by comparing the decreased NO concentration in TCM-treated group with that in LPS-stimulated group, was adopted to evaluate the anti-inflammatory property. Didox with an approximately 60 % inhibition of NO production at 100 μM was used as a positive control. The inhibitory potency of TCM extracts on NO production was ranked according to the criteria as follows: strong (MIR ≥ 80 %); moderate (80 % > MIR ≥ 50 %); weak (50 % > MIR ≥ 30 %); undetected (MIR <30 %).

Our investigation indicated that 55 TCM extracts inhibited the LPS-induced NO production with MIRs between 30.7 % and 100 % under the tested nontoxic concentrations (Table 1). Thereinto, 11 TCM extracts strongly inhibited NO production in RAW 264.7 cells (MIR ≥ 80 %), including *Artemisia argyi* (leaf, 26), *Aucklandia lappa* (root, 31), *Callicarpa macrophylla* (leaf, 35), *Chrysanthemum morifolium* (flower, 39), *Cimicifuga heracleifolia* (rhizome, 40), *Fraxinus rhynchophylla* (bark, 80), *Glycyrrhiza uralensis* (rhizome, 85), *Inula helenium* (root, 92), *Oraxylum inddicum* (seed, 123), *Physalis alkekengi* L. var. *franchetii* (calyx, 132), and *Scutellaria baicalensis* (rhizome, 168). Moreever, 25 extracts displayed moderate inhibitory effect of NO production (80 % > MIR ≥ 50 %), and 19 extracts weakly inhibited NO production (50 % > MIR ≥30 %). Inhibitory effects on NO production of 55 bioactive TCM extracts in RAW 264.7 cells have been detailedly summarized in Additional file 1: Table S1 and Figure S1.

Since oxidative stress and inflammatory response have the synergistic reactions in the pathophysiology of COPD, TCMs having dual inhibitions on the two targets are apt to be the resource for discovering lead molecules [5, 7]. Our results indicated that the extracts of *Artemisia scoparia* (aerial part, 27), *Aucklandia lappa* (root, 31), *Cassia angustifolia* (leaf, 36), *Cimicifuga heracleifolia* (rhizome, 40), *Cirsium setosum* (aerial part, 43), *Curculigo orchioides* (rhizome, 58), *Eucommia ulmoides* (root-bark, 74), *Glycyrrhiza uralensis* (rhizome, 85), *Inula helenium* (root, 92), *Ligusticum chuanxiong* (rhizome, 99), *Lithospermum erythrorhizon* (root, 103), *Morus alba* (branch, 120), *Perilla frutescens* (leaf, 128), *Physalis alkekengi* var. *franchetii* (calyx, 132), *Pogostemon cablin* (aerial part, 137), *Rabdosia rubescens* (aerial part, 154), *Rosa laevigata* (fruit, 160), *Salvia miltiorrhiza* (root and rhizome, 162), *Siegesbeckia orientalis* (aerial part, 174), and *Zanthoxylum schinifolium* (peel, 196) simultaneously inhibited oxidative stress and inflammation (Table 1 and Additional file 1: Table S1). Most of all, both QR inducing effects and NO inhibitory activities of the extracts of *Aucklandia lappa* (31), *Cimicifuga heracleifolia* (40), and *Glycyrrhiza uralensis* (85) are labelled as the level of strong. In addition, the extracts of *Inula helenium* (92) and *Physalis alkekengi* L. var. *franchetii* (132) also demonstrated the potencies that are closed to the strong level.

To our knowledge, this is the first systemic screening of QR inducing extracts from TCMs to discover TCMs with the capacity of inhibiting oxidative stress. Plenty of work on investigation of natural-derived molecules for their regulation on oxidative stress have been carried out, and acquired some active ingredients existed in above evaluated TCMs, such as andrographolide from *Andrographis paniculata* (16) [16], (*Z*)-ligustilide from *Angelica sinensis* (20) [17], dehydroglyasperin C from *Glycyrrhiza uralensis* (85) [18], isoalantolactone from *Inula helenium* (92) [19], 2’,3’-dihydroxy-4’,6’-dimethoxychalcone from *Perilla frutescens* (128) [20], oridonin from *Rabdosia rubescens* (154) [21], danshensu and tanshinone I from *Salvia miltiorrhiza* (162) [22]. These data support our observed QR inducing effects of the active TCMs. More importantly, the majority of QR inducing TCMs tested in present research have still not been phytochemically investigated through targeting oxidative stress inhibition, which affords us an opportunity to discover new lead molecules from them [23].

TCMs have been adopted for the therapy of inflammation-related diseases with a long history in China. Compared with QR inducing assay, NO inhibitory effect assay and other in vitro and in vivo anti-inflammatory models are classical and commonly adopted biological research methods, and accordingly more literatures concerning inflammation of TCMs have been published. Based on our findings, we carried out a systemic search of reported inflammation-related literatures of our observed 55 active TCM extracts, and concluded that: some TCMs have been comprehensively investigated for their anti-inflammatory property and resulted in the discovery of diverse types of natural products, covering agrimonolide from *Agrimonia pilosa* (8) [24], (-)-nyasol from *Anemarrhena asphodeloides* (17) [25], alantolactone from *Aucklandia lappa* (31) [26], berberine from *Coptis chinensis* (53) [27], forsythiaside from *Forsythia suspensa* (79) [28], resveratrol from *Polygonum cuspidatum* (141) [29], etc. Beside these comprehensively investigated molecules, a great deal of constituents have been isolated from these active TCMs, and required further confirmation of their anti-inflammatory function. Meanwhile, a number of TCMs [e.g. *Alisma orientalis* (11), *Equisetum hiemale* (72), *Cirsium setosum* (43)] have not been investigated in the field of inflammation. Significantly, little research on the therapeutic effect of COPD has been performed, and thus these active TCMs are still being researched.

In the present screening assay, we only adopted two typical markers, QR and NO, to evaluate the potential of TCMs as oxidative stress and inflammation inhibitory agents. Based on our preliminary results, active TCM extracts could be subjected to further research in the field of phytochemistry and pharmacology, however, solid evidences on their biological functions are required before a systemic investigation [14]. With regard to the inhibition on oxidative stress, the levels of endogenous glutathione (GSH) and reactive ROS, as well as the protein level of key intracellular redox-balancing protein GCLM, are suggested to be detected to estimate the intracellular redox state and antioxidant capacity when exposed to TCM extracts [30–32]. Concerning the inhibition of inflammation by the active TCMs, the levels of crucial inflammatory mediators in the COPD pathology, including TNFα, LTB4, and IL-8, should be determined to confirm their anti-inflammatory potential [33].

Additionally, the pivotal regulators for oxidative stress and inflammation should be sufficiently investigated to verify action of mechanism of the active TCMs. The transcription factor Nrf2 plays a dominant role for regulating oxidative stress. It is ubiquitously expressed in human organs, particularly rich in lung, and counteracts oxidative injury through activating intracellular redox-balancing proteins (e.g. GCLM, GST, HO-1) and up-regulating endogenous antioxidants (e.g. GSH) [11, 34]. NF-kB regulates the expression of proinflammatory genes including cytokines, chemokines, and adhesion molecules, and its inhibition therefore definitely relieves the inflammatory response of COPD [7, 35]. It has also been verified that phosphatidylinositol 3-kinase (PI3K) and mitogen-activated protein kinase (MAPK) are involved in the regulation of inflammatory response [36, 37]. Hence, the further research on active TCM extracts and purified ingredients could focus on their action of mechanism on Nrf2, NF-kB, PI3K, and MAPK signaling pathways, as well as the cross talk between these pathways.

## Conclusion

Although the present research indicates that some TCMs possessed inhibitory effects on inflammation and oxidative stress, further pharmacological investigations in vitro and in vivo models are warranted. Furthermore, bioassay-guided fractionations and identifications of active ingredients should be launched to help us illustrate the mechanism of these active species, and discover new lead molecules with unknown mechanisms and potent functions on oxidative stress- and inflammation-related diseases, especially COPD. Accordingly, these results may give new insight in research and development of COPD therapeutic agents.

## Additional file

Additional file 1: Figure S1. NADP(H): quinone oxidoreductase (QR) inducing effects of 38 bioactive TCM extracts in hepa 1c1c7 cells. The QR inducing effect was determined after 24h treatment of the hepa 1c1c7 cells in the presence or absence of tested TCMs. The data of the untreated control group was normalized as 1, and then the QR inducing activity of tested extracts was represented by the maximum folds of QR inducing activity (MQI) compared with the untreated control group. Sulforaphane (SF, 2.0 μM) was used as a positive control. The data are reported the means ± SD from three independent experiments. **Figure S2.** Inhibitory effects on NO production of 55 bioactive TCM extracts in RAW 264.7 cells. The NO concentration in the RAW 264.7 cell culture media was determined through the Griess reaction 24 h after treated in the presence or absence of tested TCMs and lipopolysaccharides (LPS, 1.0 μg/mL). Didox (100 μM) was adopted as a positive control. The data are reported the means ± SD from three independent experiments. The maximum inhibition rates (MIRs) of NO production under the untoxic tested concentration were calculated by comparing the decreased NO concentration in TCM-treated group with that in LPS-stimulated group. **Table S1.** TCM extracts with QR inducing activity and/or NO inhibitory effect. (DOCX 4312 kb)

## Abbreviations

ARE: Antioxidant response element
COPD: Chronic obstructive pulmonary disease
GCLM: Glutamate-cysteine ligase, modifier subunit
GSH: Glutathione
GST: Glutathione S transferase
HO-1: Heme oxygenase-1
IL-8: Interleukin-8
LPS: Lipopolysaccharides
LTB4: Leukotriene B4
MAPK: Mitogen-activated protein kinase
MIR: Maximum inhibition rate
MQI: Maximum folds of QR inducing activity
MTT: 3-(4,5-dimthylthiazol-2-yl)-2,5-diphenyltetrazolium bromide
NF-kB: Nuclear factor kB
Nrf2: Nuclear factor E2-related factor 2
PI3K: Phosphatidylinositol 3-kinase
QR: NADP(H):quinone oxidoreductase
ROS: Reactive oxygen species
SF: Sulforaphane
TCM: Traditional Chinese medicine
TGF-β: Transforming growth factor-β
TNFα: Tumor necrosis factor α
