# Supplementary material for: Screening of traditional Chinese medicines with therapeutic potential on chronic obstructive pulmonary disease through inhibiting oxidative stress and inflammatory response
Source: BMC Complement Altern Med. 2016 Sep 13;16(1):360. doi: 10.1186/s12906-016-1347-y (PMC5022167; doi:10.1186/s12906-016-1347-y)
Supplement: Additional file 1: Figure S1. — NADP(H): quinone oxidoreductase (QR) inducing effects of 38 bioactive TCM extracts in hepa 1c1c7 cells. The QR inducing effect was determined after 24h treatment of the hepa 1c1c7 cells in the presence or absence of tested TCMs. The data of the untreated control group was normalized as 1, and then the QR inducing activity of tested extracts was represented by the maximum folds of QR inducing activity (MQI) compared with the untreated control group. Sulforaphane (SF, 2.0 μM) was used as a positive control. The data are reported the means ± SD from three independent experiments. Figure S2. Inhibitory effects on NO production of 55 bioactive TCM extracts in RAW 264.7 cells. The NO concentration in the RAW 264.7 cell culture media was determined through the Griess reaction 24 h after treated in the presence or absence of tested TCMs and lipopolysaccharides (LPS, 1.0 μg/mL). Didox (100 μM) was adopted as a positive control. The data are reported the means ± SD from three independent experiments. The maximum inhibition rates (MIRs) of NO production under the untoxic tested concentration were calculated by comparing the decreased NO concentration in TCM-treated group with that in LPS-stimulated group. Table S1. TCM extracts with QR inducing activity and/or NO inhibitory effect. (DOCX 4312 kb) [file 12906_2016_1347_MOESM1_ESM.docx]

**Supplementary Material**

**Screening of traditional Chinese medicines with therapeutic potential on chronic obstructive pulmonary disease through inhibiting oxidative stress and inflammatory response**

Ming-Xing Zhou ^1^, Xuan Wei ^2^, Ai-Ling Li ^1^, Ai-Min Wang ^1^, Ling-Zi Lu ^1^, Yue Yang ^1^, Dong-Mei Ren ^1^, Xiao-Ning Wang ^1^, Xue-Sen Wen ^1^, Hong-Xiang Lou ^1^, Tao Shen ^1^

**Affiliation**

^1^Key Lab of Chemical Biology (MOE), School of Pharmaceutical Sciences, Shandong University, Jinan, P. R. China

^2^ School of Pharmaceutical Sciences, Shandong University of Traditional Chinese Medicine, Jinan, P. R. China

**Correspondence**

Dr. Tao Shen, School of Pharmaceutical Sciences, Shandong University, 44 West Wenhua Road, Jinan 250012, P. R. China; E-mail address: shentao@sdu.edu.cn

**Figure S1. NADP(H): quinone oxidoreductase (QR) inducing effects of 38 bioactive TCM extracts in hepa 1c1c7 cells.** The QR inducing effect was determined after 24h treatment of the hepa 1c1c7 cells in the presence or absence of tested TCMs. The data of the untreated control group was normalized as 1, and then the QR inducing activity of tested extracts was represented by the maximum folds of QR inducing activity (MQI) compared with the untreated control group. Sulforaphane (SF, 2.0 μM) was used as a positive control. The data are reported the means ± SD from three independent experiments.

**Figure S1. (Continued)**

**Figure S1. (Continued)**

**Figure S2.** **Inhibitory effects on NO production of 55 bioactive TCM extracts in RAW 264.7 cells.** The NO concentration in the RAW 264.7 cell culture media was determined through the Griess reaction 24 h after treated in the presence or absence of tested TCMs and lipopolysaccharides (LPS, 1.0 μg/mL). Didox (100 μM) was adopted as a positive control. The data are reported the means ± SD from three independent experiments. The maximum inhibition rates (MIRs) of NO production under the untoxic tested concentration were calculated by comparing the decreased NO concentration in TCM-treated group with that in LPS-stimulated group.

**Figure S2. (Continued)**

**Figure S2. (Continued)**

**Figure S2. (Continued)**

**Table S1** TCM extracts with QR inducing activity and/or NO inhibitory effect

| **No** | **Plant name** | **Part used in TCM** | **Voucher ID** | **Extract yields (%)** | **QR inducing activity (MQI)** | **Inhibition of NO production (MIR)** |
| --- | --- | --- | --- | --- | --- | --- |
| 2 | *Acanthopanax gracilistylus* W. W. Smith | Root-bark | 20150802-20-WJP | 9.8 | N/D | 68% at 100 μM |
| 3 | *Acanthopanax senticosus* (Rupr. et Maxim.) Harms | Rhizome | 20150801-8-CWJ | 7.7 | N/D | 52.0% at 200 μM |
| 8 | *Agrimonia pilosa* Ledeb. | Aerial part | 20151128-31-XHC | 9.8 | N/D | 41.2% at 200 μM |
| 10 | *Albizia julibrissin* Durazz. | Bark | 20151128-134-HHP | 8.9 | 1.64 fold at 200 μM | N/D |
| 11 | *Alisma orientalis* (Sam.) Juzep. | Root | 20151128-71-ZX | 13.6 | N/D | 34.2% at 200 μM |
| 16 | *Andrographis paniculata* (Burm.f.) Nees | Aerial part | 20151128-83-CXL | 9.9 | 2.04 fold at 200 μM | N/D |
| 17 | *Anemarrhena asphodeloides* Bge. | Rhizome | 20150802-17-ZM | 10.2 | N/D | 41.2% at 200 μM |
| 20 | *Angelica sinensis* (Oliv.) Diels | Root | 20151128-34-DG | 19.4 | 1.41 fold at 200 μM | N/D |
| 23 | *Areca catechu* L. | Fruit | 20151128-145-BL | 8.2 | 1.33 fold at 25 μM | N/D |
| 24 | *Arisaema erubescens* (Wall.) Schott | Tuber | 20151128-23-TNX | 1.2 | 1.36 fold at 200 μM | N/D |
| 26 | *Artemisia argyi* Levi. et Vant. | Leaf | 20150716-6-AY | 12.9 | N/D | 86.2% at 200 μM |
| 27 | *Artemisia scoparia* Waldst. et Kit. | Aerial part | 20151128-74-YC | 12.8 | 1.48 fold at 200 μM | 38.8% at 100 μM |
| 29 | *Atractylode lancea* (Thunb.) DC. | Rhizome | 20151128-136-CZ | 24.2 | N/D | 52.3% at 200 μM |
| 31 | *Aucklandia lappa* Decne. | Root | 20151128-15-MX | 16.3 | 2.31 fold at 25 μM | 94.7% at 25 μM |
| 35 | *Callicarpa macrophylla* Vahl | Leaf | 20151128-7-DYZZ | 4.3 | N/D | 85.8% at 200 μM |
| 36 | *Cassia angustifolia* Vahl. | Leaf | 20150802-25-FXY | 9.4 | 1.54 fold at 50 μM | 79.7% at 200 μM |
| 39 | *Chrysanthemum morifolium* Ramat. | Flower | 20150802-28-JH | 23.3 | N/D | 90.2% at 200 μM |
| 40 | *Cimicifuga heracleifolia* Kom. | Rhizome | 20151128-28-SM | 14.1 | 1.95 fold at 100 μM | 86.4% at 200 μM |
| 43 | *Cirsium setosum* (Willd.) M. Bieb. | Aerial part | 20151128-10-XJ | 12.7 | 1.51 fold at 200 μM | 38.8% at 200 μM |
| 46 | *Citrus limon* (L.) Burm. f. | Fruit | 20150716-16-NM | 19.5 | 1.79 fold at 200 μM | N/D |
| 53 | *Coptis chinensis* Franch. | Rhizome | 20150802-31-HL | 6.6 | N/D | 57.1% at 200 μM |
| 56 | *Cremastra appendiculata* (D. Don) Makino | Pseudobulb | 20151128-12-SCG | 2.6 | N/D | 61.0% at 200 μM |
| 58 | *Curculigo orchioides* Gaertn. | Rhizome | 20151128-121-XM | 4.5 | 1.57 fold at 200 μM | 47.8% at 200 μM |
| 59 | *Curcuma phaeocaulis* Val. | Rhizome | 20150801-20-EZ | 2.6 | N/D | 66.9% at 25 μM |
| 64 | *Cyperus rotundus* L. | Rhizome | 20151128-80-XF | 11.6 | 1.74 fold at 200 μM | N/D |
| 65 | *Dendrobium nobile* Lindl. | Stem | 20150802-13-MH | 9.0 | N/D | 38.9% at 200 μM |
| 68 | *Dipsacus asperoides* C. Y. Cheng et T. M. Ai | Rhizome | 20150716-13-XD | 17.5 | N/D | 38.6% at 200 μM |
| 72 | *Equisetum hiemale* L. | Aerial part | 20151128-108-MZ | 4.9 | N/D | 41.7% at 200 μM |
| 74 | *Eucommia ulmoides* Oliv. | Root-bark | 20151128-51-DZ | 8.3 | 1.56 fold at 200 μM | 62.5% at 200 μM |
| 79 | *Forsythia suspensa* (Thnub.) Vahl | Fruit | 20151128-53-LQ | 28.3 | N/D | 48.3% at 200 μM |
| 80 | *Fraxinus rhynchophylla* Hance | Bark | 20151128-86-QP | 8.0 | N/D | 90.7% at 200 μM |
| 85 | *Glycyrrhiza uralensis* Fisch. | Rhizome | 20150716-5-GC | 15.7 | 2.19 fold at 100 μM | 82.9% at 200 μM |
| 90 | *Illicium difengpi* K. I .B. et K. I. M. | Bark | 20151128-132-DFP | 1.9 | 1.52 fold at 100 μM | N/D |
| 91 | *Illicium verum* Hook. f. | Fruit | 20151128-2-BJHX | 13.3 | N/D | 55.7% at 200 μM |
| 92 | *Inula helenium* L. | Root | 20150802-5-TMX | 13.2 | 1.77 fold at 12.5 μM | 100% at 100 μM |
| 94 | *Isatis indigotica* Fort. | Leaf | 20151128-102-DQY | 13.6 | 1.66 fold at 50 μM | N/D |
| 99 | *Ligusticum chuanxiong* Hort. | Rhizome | 20151128-19-CX | 16.1 | 1.73 fold at 200 μM | 69.0% at 200 μM |
| 102 | *Lindera aggregata* (Sims) Kosterm. | Root | 20150801-15-WY | 10.7 | 1.59 fold at 200 μM | N/D |
| 103 | *Lithospermum erythrorhizon* Sieb. et Zucc. | Root | 20151128-93-ZC | 6.4 | 1.52 fold at 50 μM | 57.1% at 200 μM |
| 110 | *Lycopodium japonicum* Thunb. | Whole plant | 20151128-138-SJC | 22.3 | N/D | 53.0% at 200 μM |
| 111 | *Lycopus lucidus*Turcz. var. *hirtus* Regel | Aerial part | 20151128-70-ZL | 13.5 | N/D | 61.6% at 200 μM |
| 118 | *Misla chinensis* Maxim. | Aerial part | 20151128-81-XR | 6.9 | 1.60 fold at 100 μM | N/D |
| 120 | *Morus alba* L. | Branch | 20151128-142-SZ | 7.4 | 1.37 fold at 100 μM | 61.2% at 200 μM |
| 123 | *Oroxylum inddicum* (L.) Vent. | Seed | 20151128-26-MHD | 14.0 | N/D | 85.4% at 200 μM |
| 128 | *Perilla frutescens* (L.) Britt. | Leaf | 20150717-6-ZS | 2.8 | 1.73 fold at 200 μM | 57.8% at 200 μM |
| 129 | *Peucedanum praeruptorum* Dunn | Root | 20151128-85-QH | 20.6 | N/D | 77.6% at 200 μM |
| 132 | *Physalis alkekengi* L. var. *franchetii* (Mast.) Makino | Calyx | 20150730-2-GJD | 14.2 | 1.79 fold at 200 μM | 91.4% at 200 μM |
| 133 | *Pinellia ternata*(Thunb.) Breit. | Tuber | 20151128-130-BX | 0.8 | 1.74 fold at 200 μM | N/D |
| 137 | *Pogostemon cablin*(Blanco) Benth. | Aerial part | 20151128-16-GHX | 5.5 | 1.73 fold at 100 μM | 56.6% at 200 μM |
| 141 | *Polygonum cuspidatum* Sieb. et Zucc. | Root and rhizome | 20151128-63-HZ | 21.7 | N/D | 60.5% at 200 μM |
| 142 | *Polygonum multiflorum* Thunb. | Root | 20151128-54-HSW | 6.9 | N/D | 35.5% at 200 μM |
| 153 | *Pyrrosia sheareri* (Bak.) Ching | Leaf | 20151128-116-SW | 12.2 | 1.85 fold at 50 μM | N/D |
| 154 | *Rabdosia rubescens* (Hemsl.) Hara | Aerial part | 20151128-39-DLC | 8.4 | 1.38 fold at 100 μM | 43.2% at 200 μM |
| 156 | *Rhaponlicum uniflorum*(L.) DC. | Root | 20151128-97-LL | 4.4 | 1.54 fold at 200 μM | N/D |
| 157 | *Rheum palmatum* L. | Root and rhizome | 20151128-103-DH | 25.6 | N/D | 60.2% at 200 μM |
| 158 | *Rhodiola crenulata* (Hook. f. et Thoms.) H. Ohba | Rhizome | 20150801-5-HJT | 13.3 | N/D | 46.3% at 200 μM |
| 160 | *Rosa laevigata* Michx. | Fruit | 20151128-68-JYZ | 25.2 | 1.67 fold at 50 μM | 57.0% at 200 μM |
| 162 | *Salvia miltiorrhiza* Bge. | Root and rhizome | 20151128-29-DS | 39.7 | 1.44 fold at 100 μM | 64.5% at 200 μM |
| 164 | *Saposhnikovia divaricata* (Turcz.) Schischk. | Root | 20150802-19-FF | 15.9 | 1.95 fold at 100 μM | N/D |
| 168 | *Scutellaria baicalensis* Georgi. | Rhizome | 20150716-12-HQ | 30.2 | N/D | 87.4% at 200 μM |
| 169 | *Scutellaria barbata* D. Don | Whole plant | 20151128-35-BZL | 10.2 | N/D | 59.0% at 200 μM |
| 172 | *Senecio scandens* Buch.-Ham. | Aerial part | 20151128-14-QLG | 11.4 | N/D | 38.1% at 200 μM |
| 174 | *Siegesbeckia orientalis* L. | Aerial part | 20151128-98-XXC | 4.8 | 1.91 fold at 200 μM | 54.9% at 200 μM |
| 175 | *Siphonostegia chinensis* Benth. | Whole plant | 20151128-119-BLJM | 9.7 | N/D | 34.6% at 200 μM |
| 178 | *Sophora japonica* L. | Flower and bud | 20151128-95-HH | 37.1 | 1.44 fold at 200 μM | N/D |
| 179 | *Sparganium stoloniferum* Buch.-Ham. | Tuber | 20151128-3-SL | 4.5 | 1.57 fold at 200 μM | N/D |
| 182 | *Stephania tetrandra* S. Moore | Root | 20151128-43-FJ | 72.9 | N/D | 50.0%at 100 μM |
| 183 | *Sterculia lychnophora* Hance | Seed | 20150801-21-PDH | 3.2 | N/D | 30.7% at 200 μM |
| 184 | *Taraxacum mongolicum* Hand.-Mazz. | Whole plant | 20150716-3-PGY | 17.2 | N/D | 42.3% at 200 μM |
| 188 | *Tussilago farfara* L. | Bud | 20150802-6-KDH | 9.9 | 2.38 fold at 50 μM | N/D |
| 190 | *Usnea diffracta* Vain. | Thallus | 20150802-2-SL | 11.0 | N/D | 44.5% at 200 μM |
| 195 | *Zanthoxylum nitidum* (Roxb.) DC. | Root | 20151128-52-LMZ | 6.1 | N/D | 38.2% at 200 μM |
| 196 | *Zanthoxylum schinifolium* Sleb. et Zucc. | Peel | 20151128-48-HJ | 21.2 | 1.75 fold at 200 μM | 50.3% at 200 μM |

MQI: the maximum folds of QR inducing activity under the tested concentration; MIR: the maximum inhibition rate of NO production under the untoxic tested concentration; N/D, undetected.
